# *In Vivo* TLR9 Inhibition Attenuates CpG-Induced Myocardial Dysfunction

**DOI:** 10.1155/2013/217297

**Published:** 2013-04-10

**Authors:** O. Boehm, P. Markowski, M. van der Giet, V. Gielen, A. Kokalova, C. Brill, A. Hoeft, G. Baumgarten, R. Meyer, P. Knuefermann

**Affiliations:** ^1^Department of Anesthesiology and Intensive Care Medicine, University Hospital Bonn, Sigmund-Freud-Straße 25, 53105 Bonn, Germany; ^2^Institute of Physiology II, University of Bonn, Nussallee 11, 53115 Bonn, Germany

## Abstract

The involvement of toll-like receptor 9 (TLR9), a receptor for bacterial DNA, in septic cardiac depression has not been clarified *in vivo*. Thus, the aim of the study was to test possible TLR9 inhibitors (H154-thioate, IRS954-thioate, and chloroquine) for their ability to protect the cardiovascular system in a murine model of CpG oligodeoxynucleotide- (ODN-) dependent systemic inflammation. Sepsis was induced by i.p. application of the TLR9 agonist 1668-thioate in C57BL/6 wild type (WT) and TLR9-deficient (TLR9-D) mice. Thirty minutes after stimulation TLR9 antagonists were applied i.v. Survival was monitored up to 18 h after stimulation. Cardiac mRNA expression of inflammatory mediators was analyzed 2 h and 6 h after stimulation with 1668-thioate and hemodynamic parameters were monitored at the later time point. Stimulation with 1668-thioate induced a severe sepsis-like state with significant drop of body temperature and significantly increased mortality in WT animals. Additionally, there was a time-dependent increase of inflammatory mediators in the heart accompanied by development of septic heart failure. These effects were not observed in TLR9-D mice. Inhibition of TLR9 by the suppressive ODN H154-thioate significantly ameliorated cardiac inflammation, preserved cardiac function, and improved survival. This suppressive ODN was the most efficient inhibitor of the tested substances.

## 1. Introduction

During the course of sepsis pattern recognition receptors (PRRs) of the innate immune system such as toll-like receptors (TLRs) have been shown to play a decisive role in the disease process [1]. Toll-like receptors bind pathogen associated molecular patterns (PAMPs) originating from non-self-sources as well as damage associated molecular patterns (DAMPs) deriving from the host organism itself. In the human organism eleven TLRs are expressed, among those TLR4 the receptor for endotoxins has been described most extensively [2]. However, in the last few years another TLR has entered the focus of scientific interest: the receptor for viral and bacterial DNA TLR9. Bacterial DNA is characterized by unmethylated cytosine-phosphate-guanine oligodesoxynucleotide (CpG motifs), which are much less prevalent in mammalian DNA. CpG motifs bind specifically to TLR9. This receptor has been found to be of pivotal importance during polymicrobial sepsis [3, 4]. The reason for its relevance may lie in the common appearance of CpG motifs in all bacterial and viral DNA. Therefore, TLR9 can be regarded as a central receptor for inflammatory response in any kind of sepsis. Thus, TLR9 may be a promising target for pharmacological immune modulation.

The heart is one of the vital organs adversely affected in severe sepsis. Importantly, the presence of impaired cardiovascular function in sepsis is associated with significantly increased mortality [5, 6]. Hence, any successful intervention should aim to improve sepsis-dependent suppression of cardiovascular function.

*In vitro* TLR9 signaling induced by the application of the synthetic stimulatory oligonucleotide 1668-thioate suppressed cardiac contractility via increased iNOS expression, which could be antagonized by S-methylisothiourea (SMT). In addition, myocytes with TLR9-deficiency (TLR9-D) proved to be insensitive to stimulation with a synthetic substitute for bacterial DNA [7]. Thus, pharmacological antagonism of TLR9 might protect the cardiovascular system from the deleterious effects of bacterial DNA. Dilatation of blood vessels exposed to polymicrobial stimuli can be prevented by the synthetic ODN (H154-thioate) inhibiting TLR9 [3]. However, the *in vivo* effect of TLR9 antagonism on the heart has not yet been investigated.

Therefore, we wondered whether application of a synthetic oligodesoxynucleotide (1668-thioate) would be sufficient to depress cardiac function *in vivo*. With respect to the importance of effective inhibition of TLR signaling in sepsis, the aim of the present study was to test different TLR9 inhibitors (H154-thioate, IRS954-thioate, and chloroquine) for their effectiveness in protecting the cardiovascular system during systemic inflammation. In order to distinguish TLR9 signaling from other inflammatory pathways the specific TLR9 ligand 1668-thioate was chosen as stimulus.

## 2. Material and Methods

### 2.1. Animals

Ten-to-twelve-week-old male C57BL/6 mice were purchased from Charles River, Sulzfeld/Germany. The body weight varied from 20 to 25 g. TLR9-deficient mice (TLR9-D; backcrossed on C57BL/6 background) were kindly provided by Professor Shizuo Akira (Department of Host Defense, Research Institute for Microbial Diseases, Osaka University, Japan). All animal studies were approved by the State Office for Nature, Environment and Consumer Affairs (Landesamt für Natur, Umwelt und Verbraucherschutz) of North-Rhine Westphalia (Recklinghausen, Germany) and conformed to the guidelines for animal experimentation of the National Institute of Health (NIH Publication No. 85-23, revised 1996). For analgesia, buprenorphine was administered (0.1 mg/kg subcutaneously).

### 2.2. Dose Finding of TLR9 Antagonists

**Figure 1:**
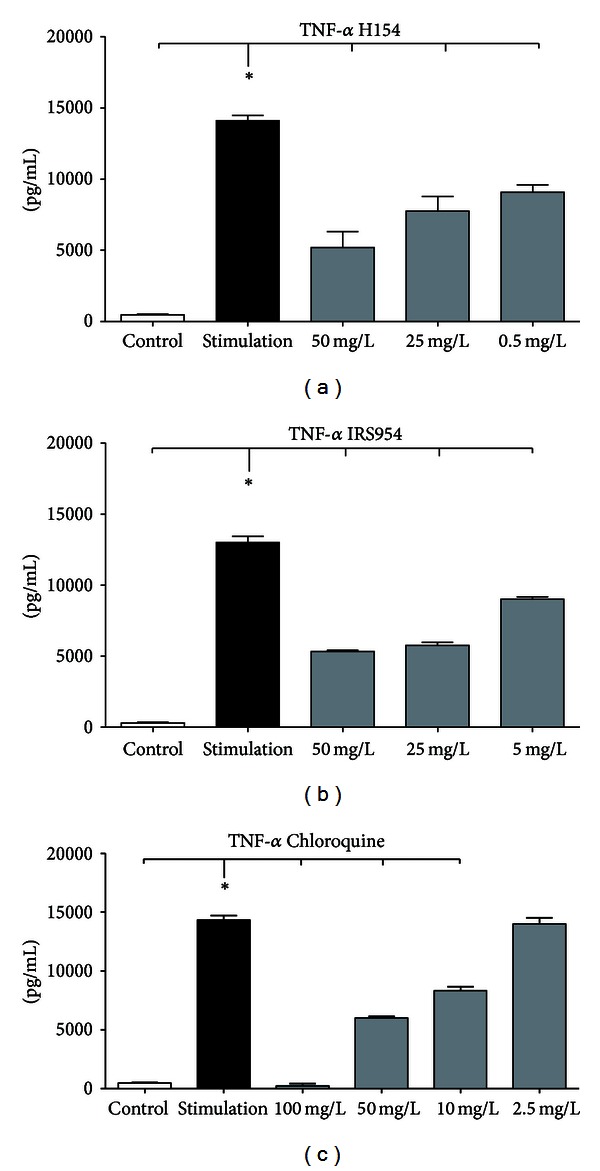
(a)–(c) *In vitro* evaluation of different doses of TLR9 inhibitors. RAW 264.7 macrophages were stimulated with feces of C57BL/6 WT mice simultaneously with different TLR9 inhibitors for 24 h and TNF-*α* protein content was monitored via ELISA (mean ± SEM; *n* = 5; *P < 0.05; *also indicates the significant group).

In a pilot study cultured macrophages of the line RAW 264.7 were stimulated with heat-inactivated feces of C57BL/6 mice for 24 h (concentration 10^6^ bacteria/mL). Thereafter, TNF-*α* protein was measured by ELISA in the supernatant of the cell culture. Stimulation led to a 30-fold increase of TNF-*α* protein compared with control (Figures 1(a)–1(c)). This increase was used to test the suppressive effect of the TLR9 inhibitors H154-thioate, IRS954-thioate, and chloroquine [8–10]. H154-thioate was applied simultaneously with the polymicrobial stimulus in three different concentrations (50 mg/L, 25 mg/L, and 0.5 mg/L). All applied concentrations of H154 were able to reduce the TNF-*α* protein significantly in a concentration-dependent manner (Figure 1(a)). Comparable experiments were performed with IRS954-thioate and chloroquine (Figures 1(b) and 1(c)). The effect of IRS954-thioate was less pronounced than that of H154-thioate. The lowest effective concentration for IRS954-thioate was 5 mg/L. Chloroquine was applied in four different concentrations (2.5, 10, 50, and 100 mg/L); the lowest effective concentration was 10 mg/L. In order to ensure efficaciousness of the antagonists a single dose of 8 mg/kg BW of H154- and IRS954-thioate and 10 mg/kg of chloroquine was applied i.v. to the animals.

### 2.3. 1668-Thioate Stimulation and Extraction of Tissue Samples

All animals were initially treated with D-GalactosamineN (D-GalN; 1 g/kg BW, Roth, Karlsruhe, Germany). NaCl 0.9% was added to attain an equal volume of 250 *μ*L. Both were injected intraperitoneally (i.p.). D-GalactosamineN was applied to slow down the hepatic degradation of CpG-ODNs [11, 12]. In control experiments D-GalN alone did not induce an inflammatory response [13]. In order to induce a systemic inflammation thirty minutes later all mice were injected i.p. with 1668-thioate (5′-TCCATGA*CG*TTCCTGATGCT; TibMolBiol, Berlin, Germany; 2 nmol/g BW = 12.1 mg/kg BW) in a total volume of 400 *μ*L PBS or with the same amount of PBS [14]. Another 30 minutes later the mice received intravenous (i.v) treatment with one of four agents: (1) a control ODN (1612-thioate; 5′-GCTAGATGTTAGCGT-3′; TibMolBiol, Berlin, Germany; 2 nmol/g BW = 9.3 mg/kg BW), (2) with H154-thioate (5′-CCTCAAGCTTGAGGGG-3′; TibMolBiol, Berlin, Germany; 8 mg/kg BW), (3) with IRS954-thioate (5′-TGCTCCTGGAGGGGTTGT-3′; TibMolBiol, Berlin, Germany; 8 mg/kg BW), (4) with chloroquine (10 mg/kg BW; Bayer Vital GmbH, Leverkusen, Germany). All i.v. injections had a volume of 200 *μ*L. Tissue was collected 2 h and 6 h after stimulation with 1668-thioate.

### 2.4. RNA-Extraction and TaqMan RT-qPCR

The mRNA expression levels of TNF-*α*, IL-6, and IL-1*β* were determined using TaqMan real-time quantitative PCR (RT-qPCR, Applied Biosystems, Darmstadt, Germany). Upon excision of the hearts total RNA was isolated (Trizol, Applied Biosystems) and first-strand cDNA was synthesized using the High-Capacity cDNA transcription kit (Applied Biosystems) with random hexameric primers according to the manufacturer's protocol. RT-qPCR was performed and analyzed with cDNA (diluted 1 : 10) on an ABI Prism 7900 Sequence Detection System and SDS2.2 Software (Applied Biosystems). Target gene expression was normalized to an internal control (glyceraldehyde-3-phosphate dehydrogenase, GAPDH). Relative RT-PCR was performed using TaqMan Gene expression Master Mix (part 4369016; Applied Biosystems) with the following primers: GAPDH (Mm99999915_g1), TNF-*α* (Mm00443258_m1), IL-1*β* (Mm99999061_g1), and IL-6 (Mm01210732_g1). All murine primers were measured using FAM TAMRA chemistry and the relative standard curve method. At the end of RT-qPCR cycle dissociation curve analysis was performed to ascertain the amplification of a single PCR product.

### 2.5. Cardiac Pressure-Volume Measurements

**Figure 2:**
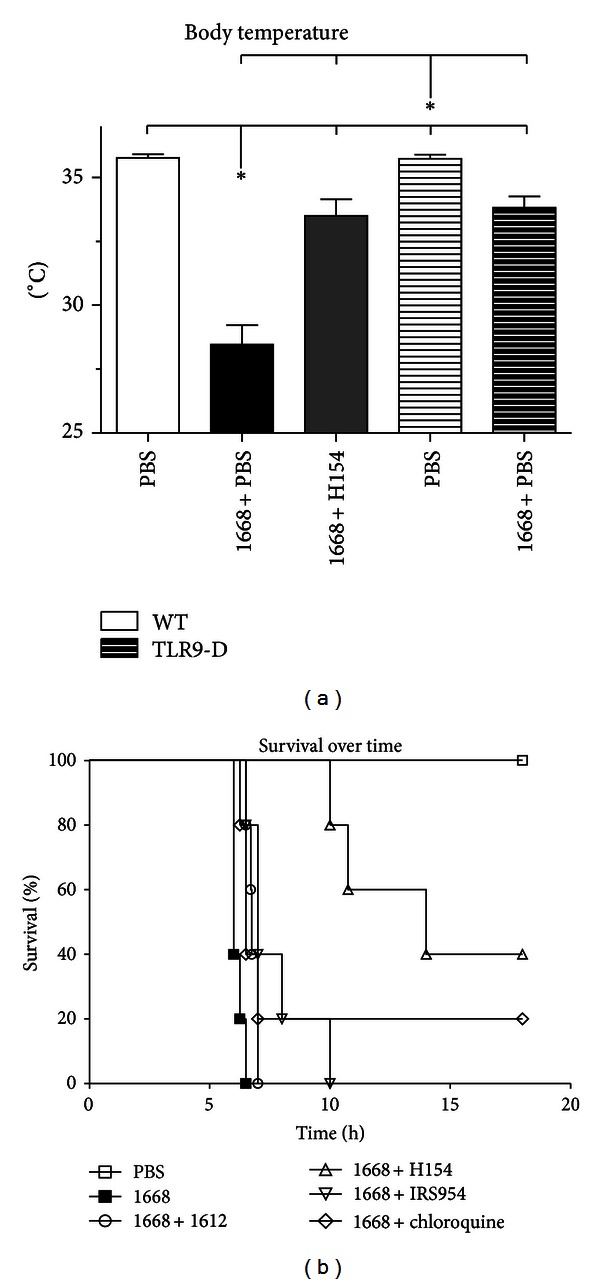
(a) Body temperature of WT- and TLR9-D mice 6 h after stimulation with the TLR9 agonist 1668-thioate. The TLR9 inhibitor H154-thioate was administered 30 min after stimulation. PBS application served as control. Bars of TLR9-D are striated (mean ± SEM; *n* = 8/group, *P < 0.05; *also indicates the significant group). (b) Survival over time of WT mice after stimulation with the TLR9 agonist 1668-thioate alone or in combination with the control ODN 1612-thioate or the TLR9 inhibitors H154- and IRS954-thioate as well as chloroquine. Inhibitors were injected i.v. 30 min after stimulation. PBS application served as control (*n* = 6/group).

Six hours after stimulation with 1668-thioate hemodynamic parameters which included left ventricular systolic pressure (LVSP), stroke volume (SV), left ventricular end-diastolic pressure (LVEDP), cardiac output (CO), and contractility indices (dP/dt_max⁡_ and dP/dt_min⁡_) were recorded using a pressure-volume catheter according to the manufacturer's manual (Millar Instruments, Houston TX). All recordings were conducted under general anesthesia with isoflurane (1 vol%). Additionally, body temperature was monitored in representative mice using a rectal probe (Figure 2(a)). For detailed descriptions see [13, 15].

### 2.6. Statistical Analysis

Statistical analysis was performed with GraphPad Prism 5.02 (GraphPad Software Inc., San Diego, USA). Significance testing included one-way ANOVA followed by Newman-Keuls *post hoc* analysis. Comparative analysis of survival was performed using the Kaplan-Meier method. Statistical significance was determined using the log-rank test. Differences were considered significant at *P* < 0.05. Data are reported as means and standard error of the mean (SEM).

## 3. Results

Clinical appearance as well as body temperature was investigated in WT and TLR9-D mice up to 18 h after 1668-thioate stimulation. In addition, survival was monitored in all groups of stimulated WT mice. After only 2 h WT mice started to display sepsis-like symptoms such as ruffled fur, food refusal, and limited ability to respond to external stimuli. In the following four hours sepsis-like symptoms worsened. Neither the specific TLR9 antagonist IRS954 nor the unspecific TLR9 inhibitor chloroquine improved the clinical appearance of WT mice stimulated with 1668-thioate *in vivo*. However, the application of H154-thioate improved clinical behavioral patterns obviously: none of the mice in this group showed external signs of systemic inflammation. In accordance with this TLR9-D mice also did not exhibit any clinical symptoms.

Six hours after 1668-thioate stimulation body temperature in both WT and TLR9-D mice significantly decreased; however, body temperature in TLR9-D mice remained above that of WT mice (Figure 2(a)). Interestingly, WT mice cotreated with H154-thioate presented nearly the same temperature as TLR9-D mice (Figure 2(a)). These findings concur with the observation that all WT animals treated with 1668-thioate were dead shortly after 6 hours. Comparable mortality was observed in animals treated with 1668-plus 1612-thioate. In contrast, the use of any of the three TLR9 antagonists prolonged survival. H154-thioate proved to be the most effective substance improving survival by 40% (Figure 2(b)). Furthermore, all TLR9-D animals survived longer than 18 hours after 1668-thioate application (data not included in Figure 2(b)).

**Figure 3:**
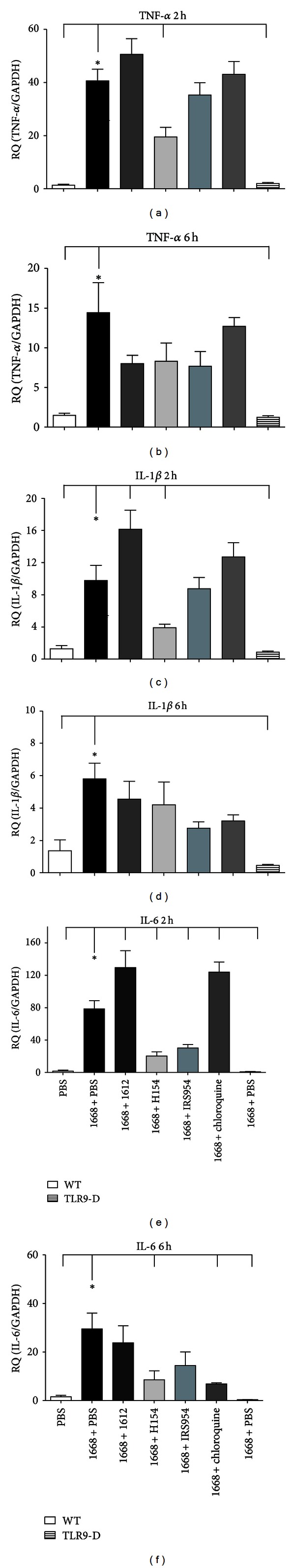
(a)–(f) RT-qPCR of mRNA expression of proinflammatory cytokines TNF-*α*, IL-1*β*, and IL-6 in the hearts of WT and TLR9-D mice measured 2 and 6 h after application of 1668-thioate. The control ODN 1612-thioate or the TLR9 inhibitors H154- and IRS954-thioate as well as chloroquine were injected 30 min after stimulation. PBS application served as control, and TLR9-D animals (last bar) were stimulated with 1668-thioate + PBS as negative control (mean ± SEM; *n* = 8/group, *P < 0.05; *also indicates the significant group).

Two hours and six hours after 1668-thioate stimulation mediators of inflammation (TNF-*α*, IL-1*β*, IL-6) were monitored in cardiac tissue (Figures 3(a)–3(f)). In WT animals, 1668-thioate challenge resulted in a significant upregulation of all three proinflammatory cytokines at both time points with maximal expression at 2 h (Figures 3(a), 3(c), and 3(e)). The additional application of the control oligonucleotide 1612-thioate led to a further increase in IL-1*β* and IL-6 mRNA expression at 2 h (Figures 3(c) and 3(e)). Four hours later, all three cytokines had decreased by at least 40% (Figures 3(b), 3(d), and 3(f)). The application of H154-thioate diminished the 1668-thioate-dependent cytokine increase of all three mediators 2 h after stimulation. After 6 h this suppression was no longer detectable in neither TNF-*α* nor Il-1*β* (Figures 3(b) and 3(d)). Neither IRS954-thioate nor chloroquine suppressed cardiac TNF-*α* or Il-1*β* mRNA expression at any time point (Figures 3(a)–3(d)). However, there was a significant downregulation of cardiac IL-6 mRNA expression in the 1668-thioate + IRS954-thioate group 2 h after stimulation (Figure 3(e)). Chloroquine application induced a biphasic response of IL-6, enhancing its expression at 2 h and diminishing it at 6 h (Figures 3(e) and 3(f)). In cardiac tissue of TLR9-D animals, application of 1668-thioate did not influence the mRNA expression of the investigated mediators. In clinical symptoms and survival as well as in cytokine mRNA expression H154-thioate proved to be the most potent inhibitor of TLR9 signaling. Therefore only this TLR9 antagonist was tested with respect to cardiac function.

**Figure 4:**
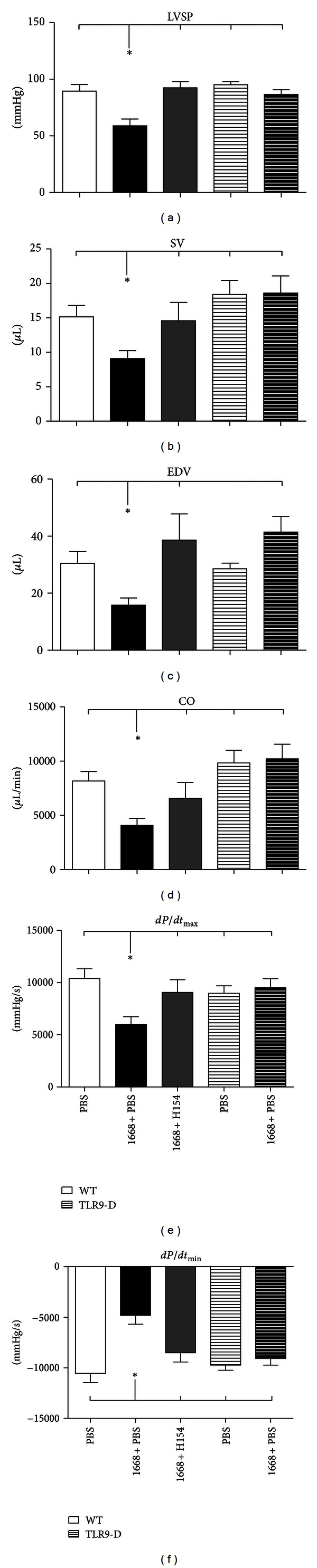
(a)–(f) Hemodynamic parameters, (a) left ventricular systolic pressure (LVSP), (b) stroke volume (SV), (c) end-diastolic volume (EDV), (d) cardiac output (CO), (e) velocity of pressure increase (dP/dt_max⁡_), and (f) velocity of pressure decrease (dP/dt_min⁡_) monitored with a pressure-volume catheter 6 h after stimulation with the TLR9 agonist 1668-thioate. Bars of TLR9-D are striated (mean ± SEM; *n* = 8/group, *P < 0.05; *also indicates the significant group).

Parameters of cardiovascular performance monitored with a pressure-volume catheter in WT as well as in TLR9-D mice 6 h after stimulation with 1668-thioate are given in Figures 4(a)–4(f). There was a significant impairment of all measured functional parameters in stimulated WT mice compared to PBS controls. In agreement with cytokine mRNA expression, additional application of H154-thioate significantly improved LV function compared to mice treated with 1668-thioate + PBS. In detail, heart rate (HR; data not shown), left ventricular systolic pressure (LVSP), stroke volume (SV), cardiac output (CO), end-diastolic volume (EDV), velocity of pressure increase (dP/dt_max⁡_), and velocity of pressure decrease (dP/dt_min⁡_) were improved by H154-thioate in comparison to 1668-thioate + PBS (Figures 4(a)–4(f)). Deficiency for TLR9 entirely prevented any deterioration of cardiac function (Figures 4(a)–4(f)).

## 4. Discussion

This study aimed to extend the *in vitro* finding that TLR9 stimulation decreases cardiomyocyte contractility to the *in vivo* setting [7]. To our knowledge the present results are first to formally demonstrate that specific TLR9 stimulation with 1668-thioate lowers hemodynamic parameters *in vivo* in a murine model of systemic inflammation. The second attempt of the present investigation was to test possible TLR9 inhibitors (H154-thioate, IRS954-thioate, and chloroquine) for their cardioprotective properties. The three tested inhibitors exhibited differential potencies; that is, H154-thioate proved to be the most effective substance.

1668-thioate has been applied in various experimental settings for the induction of a TLR9-dependent systemic inflammation as well as organ dysfunction [7, 11, 14, 16, 17]. The effect of TLR9-dependent systemic inflammation as well as organ dysfunction seems to be dose dependent as a low concentration (0.25 nmol/g) can serve as a mild stimulus for cardiac preconditioning [13], whereas higher concentrations in the range from 0.5 to 1 nmol/g induce sepsis-like inflammation [7, 17]. In order to warrant clear changes in cardiac function the formerly applied dose of 1 nmol/g was doubled to 2 nmol/g BW in the current study. This challenge resulted in serious inflammation as well as in drastic myocardial depression. Furthermore, the relatively high dose of 1668-thioate applied here seemed to be meaningful with respect to testing antagonizing strategies. 

Thirty minutes after 1668-thioate stimulation mice received i.v. treatment either with a control ODN (1612-thioate) or with one of the three inhibitors. The rationale for the i.v. application of the antagonists was to avoid a possible direct interaction between stimulating and inhibitory substances in the peritoneal cavity. Furthermore, the delayed injection of the antagonists intravenously mimics best the clinical situation.

Specific TLR9 stimulation led to a time-dependent upregulation of cardiac TNF-*α*, IL-1*β*, and IL-6 mRNA expression with a peak 2 h after stimulation and a reduced level 4 h later. This time course as well as the TLR9-dependent cytokine induction confirms earlier findings of our group [7]. An interesting new aspect is that costimulation with 1612-thioate increased the expression of all three pro-inflammatory mediators at 2 h, reaching significance in the case of IL-1*β* and IL-6 mRNA. It has been shown that application of 1612-thioate alone failed to induce cytokine expression [7]. However, prestimulation with 1668-thioate may sensitize the organism to succeeding stimuli in a way that originally inert substances (1612-thioate) are able to act as second hits, thereby further increasing the inflammatory response. A possible explanation for the enhanced mediator response may lie in the weak immune stimulatory properties of the charged phosphorothioate backbone of 1612-thioate [18].

H154-thioate was the sole antagonist, which significantly depressed the upregulation of the three tested cytokines 2 h after stimulation with 1668-thioate. In contrast, IRS954-thioate reduced upregulation of IL-6 mRNA only. Chloroquine failed to develop any protective influence at this time point, but even enhanced the expression of IL-6 (Figures 3(a), 3(c), and 3(e)). Four hours later, the expression of the three cytokines had fallen to at least 60%, which was still significantly above the PBS and TLR9-D controls. Also in the groups with additional application of antagonists the absolute levels of cytokine expression had fallen. Significant antagonistic effects appeared only for the combinations of IL-6 and H154-thioate as well as IL-6 and chloroquine (Figures 3(b), 3(d), and 3(f)). Taken together, H154-thioate had the strongest effect of the three inhibitors.

TLR9 is found in the endosomal compartment. Ligands applied from the extracellular space have to be internalized and transported to the endosomal compartment. Chloroquine has been shown to disrupt this vesicle trafficking and acidification, which can explain the inhibition of the innate immune response [19]. These data were mainly derived from *in vitro* experiments. However, there are reports that chloroquine is also effective *in vivo* [8, 20]. In a model of polymicrobial sepsis, chloroquine improved survival and reduced renal injury as well as systemic inflammation [20]. With respect to survival these findings appear to contradict ours. However, there are differences between the model in this study and that of Yasuda et al. [20]. We applied a specific TLR9 stimulus in a concentration high enough to induce a drastic systemic inflammation causing death of 100% of the animals shortly after 6 h, whereas Yasuda et al. applied cecal ligation and puncture (CLP) with a mortality of 50% after more than 48 h; that is, the CLP-dependent inflammation was less severe than the one in the present study. In addition to the inhibition of vesicle trafficking, it has been proposed that chloroquine decreases TLR9 protein. Thus, TLR9 signaling itself may be a major target for the protective actions of chloroquine [8]. This downregulation of TLR9 protein was detected 18 h after application of chloroquine [20]; this mechanism is possibly too slow to develop a beneficial influence in our model. In another attempt, Hong et al. demonstrated that chloroquine at a dose of 30 mg/kg BW could protect mice from lethal challenge by 1668-thioate, whereas chloroquine at a dose of 25 mg/kg BW could decrease serum TNF-*α* and IL-6 in rats injected with sublethal doses of CpG-ODN [8]. In our hands, preliminary tests of a high chloroquine dose of 30 mg/kg BW caused a 100% mortality immediately after i.v. injection (data not shown). We therefore stayed with a dose of 10 mg/kg BW as explained in Material and Methods section. This divergence in chloroquine sensitivity may be attributed to differences in mouse strains (BALB/C versus C57BL/6N mice) [8]. Taken together, in our model of CpG-induced sepsis the protective abilities of chloroquine could not be confirmed.

Synthetic oligonucleotides containing inhibitory properties such as H154-thioate and IRS954-thioate are frequently used in experimental studies to suppress TLR signaling [21, 22]. Several mechanisms of action seem to contribute to the suppressive activity. Pisetsky's group described synthetic oligonucleotides containing poly-G sequences, which block bacterial DNA-induced activation [23, 24]. The inhibition is seen at relatively high micromolar concentrations. Furthermore, it has been suggested that suppressive ODNs interfere with the phosphorylation of signal transducer and activator of transcriptions 1 and 4 (STAT1 and STAT4, resp.), thereby blocking inflammation mediated by STAT-associated signaling cascades [25]. This interaction may be highly specific because suppressive ODNs do not bind to other molecules in the NF*κ*B and MAPK regulatory cascade, and control ODNs do not bind to STAT1 or STAT4 [25, 26]. Furthermore, *in vitro* studies have demonstrated that inhibitory ODNs preferentially bind to the C-terminal of TLR9, competing for CpG-ODN binding [27]. With respect to the structural details of inhibitory ODNs, Ashman et al. demonstrated *in vitro* that a specific motif (CCx(not-C)(not-C)xxGGG (x = any base)) provides the sequences required to block TLR9 in human B cells and HEK cells transfected with human TLR9. Extending the sequence by four to five bases at the 5′ end enhanced activity, even more so when a phosphorothioate backbone replaced the native phosphodiester backbone [28] (for further details, see [25, 26]). In addition to the molecular composition of an inhibitory ODN also the targeted cell type has been shown to influence the inhibitory potency of the respective ODN; that is, some inhibitory sequences that function well in macrophages have been shown to be inefficient in B cells [29]. Taken together, the workings of molecular blocking mechanisms of inhibiting ODN have not yet been unraveled; differences in the potency of H154-thioate and IRS954-thioate up to now cannot be attributed to their molecular structure.

Contractile activity of cardiomyocytes *in vitro* is depressed by 1668-thioate stimulation [7]. *In vitro* findings are generated in a highly controlled environment, which is not the case in living organisms. Therefore, transfer from *in vitro* to *in vivo* cannot be assumed to be self-evident. Thus, the presented cardiodepressive effect of 1668-thioate stimulation *in vivo* enhances our knowledge of the understanding of TLR9 stimulation. This is further supported by the total insensitivity of TLR9-D mice to this stimulation. To our knowledge, this study is the first demonstration that specific TLR9 stimulation with 1668-thioate lowers hemodynamic parameters *in vivo* in a murine model of systemic inflammation. In our experimental setting H154-thioate proved to be the strongest inhibitor of all tested substances. Therefore, analyses of hemodynamic parameters were only performed with this inhibitor. There was a significant impairment of all measured functional parameters in stimulated WT mice compared to PBS controls. In agreement with cytokine mRNA expression, additional application of H154-thioate significantly improved LV function when compared to mice treated with 1668-thioate + PBS. Specifically, heart rate, left ventricular systolic pressure, stroke volume, cardiac output, end-diastolic volume, velocity of pressure increase, and velocity of pressure decrease were improved by H154-thioate in comparison to 1668-thioate + PBS (Figures 4(a)–4(f)). Deficiency for TLR9 entirely prevented any deterioration of cardiac function (Figures 4(a)–4(f)). Our finding that H154-thioate protected the heart against TLR9-stimulation complements the earlier observation that H154-thioate prevented TLR9-dependent vascular relaxation [3]. In fact, preserved vascular resistance may contribute to improved hemodynamic performance found here.

The importance of TLR9 signaling in polymicrobial infection has already been shown by others and by our group [3, 4]. A possible reason for this may lie in the ability of TLR9 to bind the DNA of Gram-positive as well as Gram-negative bacteria and thus to react to a broad range of stimuli [3, 30, 31]. The incidence of polymicrobial infections is relatively high in clinically severe sepsis [32]. Therefore, the strategy of specific TLR4-blocking may be inferior to that of TLR9-blocking. This may have contributed to the disappointing results of the application of Eritoran (E5564), a TLR4 antagonist, evaluated in the ACCESS (A Controlled Comparison of Eritoran and Placebo in Patients with Severe Sepsis) trial, a global, randomized, double-blind, placebo-controlled Phase III study [33].

Taken together, here we show that systemic TLR9 stimulation is able to depress cardiac function *in vivo* and that a pharmacological intervention is possible and may be a promising strategy for human clinical trials in future.

## References

[1] Mohan C, Zhu J (2010). Toll-like receptor signaling pathways—therapeutic opportunities. *Mediators of Inflammation*.

[2] Seeley EJ, Matthay MA, Wolters PJ (2012). Inflection points in sepsis biology: from local defense to systemic organ injury. *American Journal of Physiology*.

[3] Ehrentraut SF, Dorr A, Ehrentraut H (2012). Vascular dysfunction following polymicrobial sepsis: role of pattern recognition receptors. *PloS One*.

[4] Plitas G, Burt BM, Nguyen HM, Bamboat ZM, DeMatteo RP (2008). Toll-like receptor 9 inhibition reduces mortality in polymicrobial sepsis. *Journal of Experimental Medicine*.

[5] Parrillo JE, Parker MM, Natanson C (1990). Septic shock in humans. Advances in the understanding of pathogenesis, cardiovascular dysfunction, and therapy. *Annals of Internal Medicine*.

[6] Zanotti-Cavazzonia SL, Hollenberg SM (2009). Cardiac dysfunction in severe sepsis and septic shock. *Current Opinion in Critical Care*.

[7] Knuefermann P, Schwederski M, Velten M (2008). Bacterial DNA induces myocardial inflammation and reduces cardiomyocyte contractility: role of Toll-like receptor 9. *Cardiovascular Research*.

[8] Hong Z, Jiang Z, Liangxi W (2004). Chloroquine protects mice from challenge with CpG ODN and LPS by decreasing proinflammatory cytokine release. *International Immunopharmacology*.

[9] Yamada H, Ishii KJ, Klinman DM (2004). Suppressive oligodeoxynucleotides inhibit CpG-induced inflammation of the mouse lung. *Critical Care Medicine*.

[10] Barrat FJ, Meeker T, Gregorio J (2005). Nucleic acids of mammalian origin can act as endogenous ligands for Toll-like receptors and may promote systemic lupus erythematosus. *Journal of Experimental Medicine*.

[11] Sparwasser T, Miethke T, Lipford G (1997). Bacterial DNA causes septic shock. *Nature*.

[12] Peter M, Bode K, Lipford GB, Eberle F, Heeg K, Dalpke AH (2008). Characterization of suppressive oligodeoxynucleotides that inhibit Toll-like receptor-9-mediated activation of innate immunity. *Immunology*.

[13] Velten M, Duerr GD, Pessies T (2012). Priming with synthetic oligonucleotides attenuates pressure overload-induced inflammation and cardiac hypertrophy in mice. *Cardiovascular Research*.

[14] Knuefermann P, Baumgarten G, Koch A (2007). CpG oligonucleotide activates Toll-like receptor 9 and causes lung inflammation *in vivo*. *Respiratory Research*.

[15] Pacher P, Nagayama T, Mukhopadhyay P, Bátkai S, Kass DA (2008). Measurement of cardiac function using pressure-volume conductance catheter technique in mice and rats. *Nature Protocols*.

[16] Ehrentraut H, Meyer R, Schwederski M (2011). Systemically administered ligands of toll-like receptor 2, -4, and -9 induce distinct inflammatory responses in the murine lung. *Mediators of Inflammation*.

[17] Sparwasser T, Miethke T, Lipford G (1997). Macrophages sense pathogens via DNA motifs: induction of tumor necrosis factor-*α*-mediated shock. *European Journal of Immunology*.

[18] Krieg AM, Yi AK, Matson S (1995). CpG motifs in bacterial DNA trigger direct B-cell activation. *Nature*.

[19] Kužnik A, Benčina M, Švajger U, Jeras M, Rozman B, Jerala R (2011). Mechanism of endosomal TLR inhibition by antimalarial drugs and imidazoquinolines. *Journal of Immunology*.

[20] Yasuda H, Leelahavanichkul A, Tsunoda S (2008). Chloroquine and inhibition of Toll-like receptor 9 protect from sepsis-induced acute kidney injury. *American Journal of Physiology*.

[21] Pawar RD, Ramanjaneyulu A, Kulkarni OP, Lech M, Segerer S, Anders HJ (2007). Inhibition of Toll-like receptor-7 (TLR-7) or TLR-7 plus TLR-9 attenuates glomerulonephritis and lung injury in experimental lupus. *Journal of the American Society of Nephrology*.

[22] Zeuner RA, Ishii KJ, Lizak MJ (2002). Reduction of CpG-induced arthritis by suppressive oligodeoxynucleotides. *Arthritis and Rheumatism*.

[23] Halpern MD, Pisetsky DS (1995). *In vitro* inhibition of murine IFN*γ*, production by phosphorothioate deoxyguanosine oligomers. *Immunopharmacology*.

[24] Pisetsky DS, Reich CF (2000). Inhibition of murine macrophage IL-12 production by natural and synthetic DNA. *Clinical Immunology*.

[25] Klinman DM, Tross D, Klaschik S, Shirota H, Sato T (2009). Therapeutic applications and mechanisms underlying the activity of immunosuppressive oligonucleotides. *Annals of the New York Academy of Sciences*.

[26] Shirota H, Gursel I, Gursel M, Klinman DM (2005). Suppressive oligodeoxynucleotides protect mice from lethal endotoxic shock. *Journal of Immunology*.

[27] Avalos AM, Ploegh HL (2011). Competition by inhibitory oligonucleotides prevents binding of CpG to C-terminal TLR9. *European Journal of Immunology*.

[28] Ashman RF, Goeken JA, Latz E, Lenert P (2011). Optimal oligonucleotide sequences for TLR9 inhibitory activity in human cells: lack of correlation with TLR9 binding. *International Immunology*.

[29] Lenert PS (2010). Classification, mechanisms of action, and therapeutic applications of inhibitory oligonucleotides for toll-like receptors (TLR) 7 and 9. *Mediators of Inflammation*.

[30] Boyd JH (2012). Toll-like receptors and opportunities for new sepsis therapeutics. *Current Infectious Disease Reports*.

[31] Feng Y, Zou L, Zhang M, Li Y, Chen C, Chao W (2011). MyD88 and Trif signaling play distinct roles in cardiac dysfunction and mortality during endotoxin shock and polymicrobial sepsis. *Anesthesiology*.

[32] Annane PD, Bellissant PE, Cavaillon JM (2005). Septic shock. *The Lancet*.

[33] Bennett-Guerrero E, Grocott HP, Levy JH (2007). A Phase II, double-blind, placebo-controlled, ascending-dose study of eritoran (E5564), a lipid a antagonist, in patients undergoing cardiac surgery with cardiopulmonary bypass. *Anesthesia and Analgesia*.

